# Media optimization for SHuffle T7 *Escherichia coli* expressing SUMO-Lispro proinsulin by response surface methodology

**DOI:** 10.1186/s12896-021-00732-4

**Published:** 2022-01-03

**Authors:** Aida Bakhshi Khalilvand, Saeed Aminzadeh, Mohammad Hossein Sanati, Fereidoun Mahboudi

**Affiliations:** 1 Bioprocess Engineering Group, Institute of Industrial and Environmental Biotechnology, National Institute of Genetic Engineering and Biotechnology (NIGEB), Tehran, Iran; 2 Medical Genetics Group, Institute of Medical Biotechnology, National Institute of Genetic Engineering and Biotechnology (NIGEB), Tehran, Iran; 3 Biotechnology Research Center, Pasteur Institute of Iran, Tehran, Iran

**Keywords:** CCD, *E. coli*, Medium composition, PBD, RSM, SHuffle

## Abstract

**Background:**

SHuffle is a suitable *Escherichia coli* (*E. coli*) strain for high yield cytoplasmic soluble expression of disulfide-bonded proteins such as Insulin due to its oxidative cytoplasmic condition and the ability to correct the arrangement of disulfide bonds. Lispro is an Insulin analog that is conventionally produced in *E. coli* as inclusion bodies (IBs) with prolonged production time and low recovery. Here in this study, we aimed to optimize cultivation media composition for high cell density fermentation of SHuffle T7 *E. coli* expressing soluble Lispro proinsulin fused to SUMO tag (SU-INS construct) to obtain high cell density fermentation.

**Results:**

Factors including carbon and nitrogen sources, salts, metal ions, and pH were screened via Plackett–Burman design for their effectiveness on cell dry weight (CDW) as a measure of cell growth. The most significant variables of the screening experiment were Yeast extract and MgCl_2_ concentration, as well as pH. Succeedingly, The Central Composite Design was utilized to further evaluate and optimize the level of significant variables. The Optimized media (OM-I) enhanced biomass by 2.3 fold in the shake flask (2.5 g/L CDW) that reached 6.45 g/L (2.6 fold increase) when applied in batch culture fermentation. The efficacy of OM-I media for soluble expression was confirmed in both shake flask and fermentor.

**Conclusion:**

The proposed media was suitable for high cell density fermentation of *E. coli* SHuffle T7 and was applicable for high yield soluble expression of Lispro proinsulin.

**Supplementary Information:**

The online version contains supplementary material available at 10.1186/s12896-021-00732-4.

## Background

Lispro, produced by Eli Lilly, is the first rapid-acting insulin analog approved for human use in 1996 [1]. This analog is suitable for post-prandial injections and overall glycemic control in insulin-dependent diabetic patients due to its accelerated action profile [2]. Lispro possesses the same pharmaceutical properties as Regular human insulin with equal molecular weight, and 1 unit of Lispro insulin has the same blood glucose-lowering effect compared to Regular human insulin. However, they have differing pharmacodynamics and pharmacokinetics. Lispro is suitable for post-prandial administration because it embarks its action after 5–15 min after injection. Regular insulin has a slower action profile and must be administrated 30–45 min before meals [1, 3]. *Escherichia coli* (*E. coli*) is the principal host strain for Lispro production [4]. According to the prone-to-aggregate nature of insulin molecule as a two-chained disulfide-bonded peptide, inclusion body (IB) formation in its heterologous expression in *E. coli*’s reducing cytoplasmic environment is inevitable [5]. However, this poses several challenges during its production procedure.

Various approaches are adopted to express soluble and active recombinant proteins in *E. coli*, such as applying solubilizing fusion tags and engineered host strains. One of the most efficient approaches is to utilize fusion tags such as small ubiquitin-related modifier (SUMO) [6]. Another strategy to increase soluble protein expression yield in *E. coli* is to employ engineered host strains with the oxidative cytoplasmic environment that is more suitable for disulfide bond formation. Origami and SHuffle strains of *E. coli* are deficient for genes responsible for cytoplasm’s reducing condition, including *trxB* (Thioredoxin reductase) and *gor* (Glutathione reductase). Thus, these strains are by far more efficient for disulfide bond formation [7]. Moreover, the SHuffle strain expresses a cytoplasmic copy of disulfide bond isomerase (DsbC), a chaperone with the ability to correct the arrangement of disulfide bonds, and therefore, minimizes the formation of IBs [8].

Besides, the chemical and nutritional components of the cultivation medium can directly affect the host cell growth during target protein synthesis [9]. Several elements may control cell growth, such as Carbon (C) and Nitrogen (N) sources, metal ions, and the medium pH. Thus, it is essential to utilize the optimum culture composition to obtain a high yield of recombinant protein [10]. The number of contributing factors is high, and thus, it is a laborious and time-consuming task to examine the effect of each level of each variable one by one via the One-factor-at-a-time approach (OFAT). Not to mention that these factors may have dependent or either independent effects or interactive influence on responses that this strategy fails to analyze. However, the factorial approach examines all levels of all factors simultaneously to determine their independent effects and their interactions [11]. Design of experiment (DoE) is a statistical tool that examines factors and their different levels simultaneously by a reduced number of experiments via fractional factorial models such as response surface methodology (RSM) to evaluate more relevant interactions among variables [12].

We aimed to optimize culture media composition to increase the biomass of *E. coli* SHuffle T7 expressing SUMO-Lispro proinsulin (SU-INS) via DoE methods. The screening experiment was carried out for several culture components by Plackett–Burman Design (PBD) to evaluate their influence on SU-INS SHuffle T7 growth. Significant factors were optimized by RSM Central Composite Design (CCD) to obtain the optimum culture media composition. Afterward, optimized media was applied in the shake flask and fermentor to evaluate the soluble expression of the fusion protein and the overall reproducibility of the suggested optimal media composition.

## Results

### Culture media optimization

#### Factor screening by Plackett–Burman design

Eleven factors were evaluated for their effectiveness on bacterial growth and 20 experiments were designed by Minitab18.1.0 Software. By the end of the experiments, final cell density was measured (g/L CDW) and reported in the response column of PBD (Table 1).

**Table 1:** Generated experimental runs for factor screening via PBD and corresponding responses

Run	N Type	N (1–4%)	Yeast extract (1–4%)	pH (6–8)	Buffer	NaCl (0-17 mM)	KCl (0-10 mM)	MgCl_2_ (5-15 mM)	MgSO_4_ (5-15 mM)	Glycerol (0–5%)	Glucose (0-10 mM)	CDW (g/L)
1	Peptone	2.5	2.5	7	+	8.5	5	10	10	2.5	10	2.18
2	Tryptone	4	4	8	+	17	10	5	15	0	0	2.15
3	Peptone	4	1	8	−	0	10	5	5	0	20	2.1
4	Peptone	2.5	2.5	7	−	8.5	5	10	10	2.5	10	2.04
5	Tryptone	1	1	8	−	17	0	15	15	0	20	1.98
6	Peptone	1	4	8	+	17	0	5	5	5	20	1.95
7	Peptone	4	1	8	+	0	0	15	15	5	0	1.95
8	Peptone	2.5	2.5	7	−	8.5	5	10	10	2.5	10	1.87
9	Tryptone	2.5	2.5	7	+	8.5	5	10	10	2.5	10	1.87
10	Tryptone	2.5	2.5	7	+	8.5	5	10	10	2.5	10	1.84
11	Tryptone	2.5	2.5	7	−	8.5	5	10	10	2.5	10	1.82
12	Peptone	1	1	6	−	17	10	5	15	5	0	1.8
13	Tryptone	2.5	2.5	7	−	8.5	5	10	10	2.5	10	1.8
14	Peptone	2.5	2.5	7	+	8.5	5	10	10	2.5	10	1.78
15	Tryptone	1	1	6	+	0	0	5	5	0	0	1.77
16	Tryptone	1	4	8	−	0	10	15	5	5	0	1.69
17	Tryptone	4	1	6	+	17	10	15	5	5	20	1.35
18	Peptone	1	4	6	+	0	10	15	15	0	20	1.2
19	Tryptone	4	4	6	−	0	0	5	15	5	20	0.94
20	Peptone	4	4	6	−	17	0	15	5	0	0	0.42

**Fig. 1:**
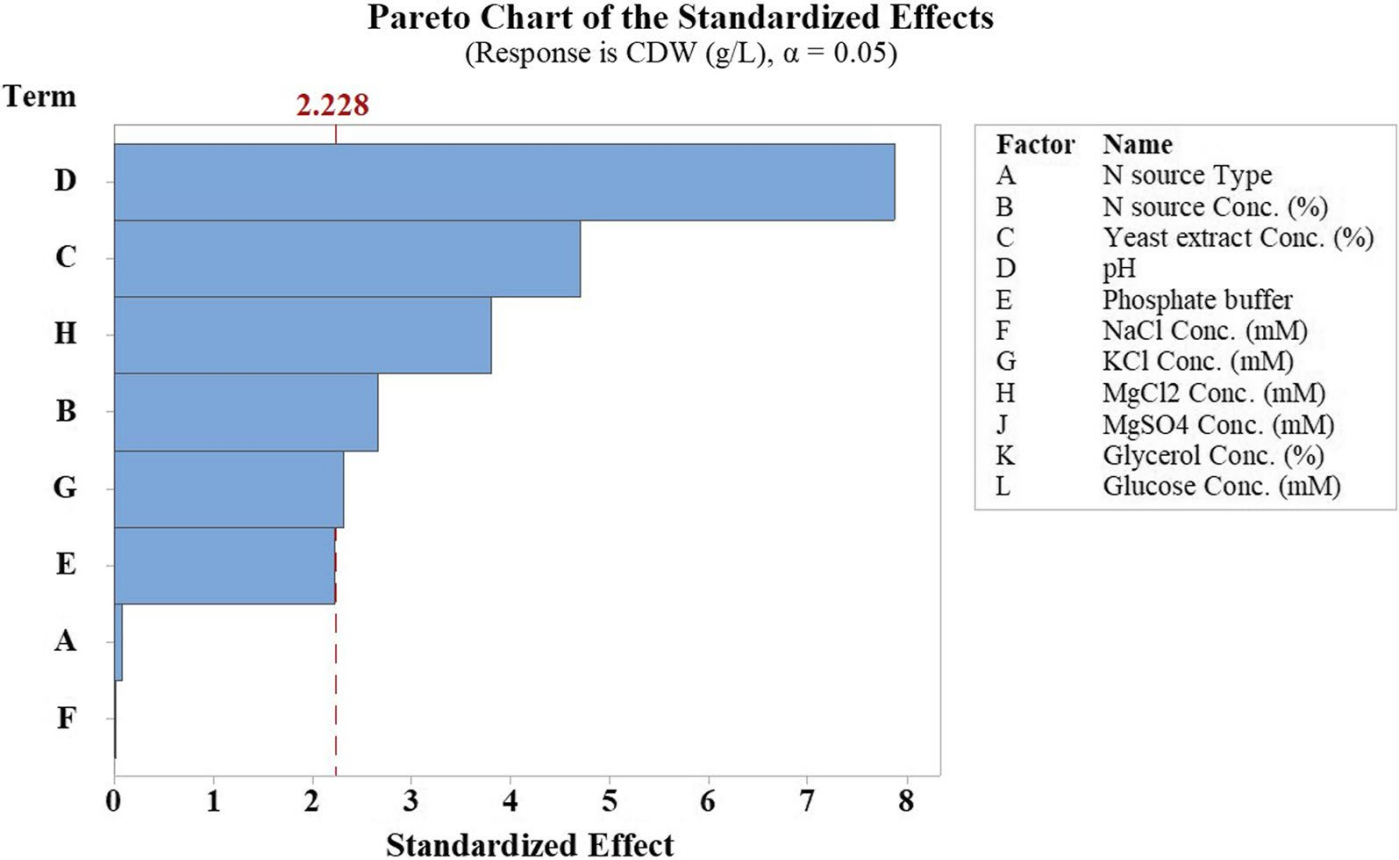
Pareto chart of Standardized effects generated by PBD from screening analyses. Statistically significant factors (*p* value < 0.05) are denoted with effect values larger than reference Line (2.228)

**Fig. 2:**
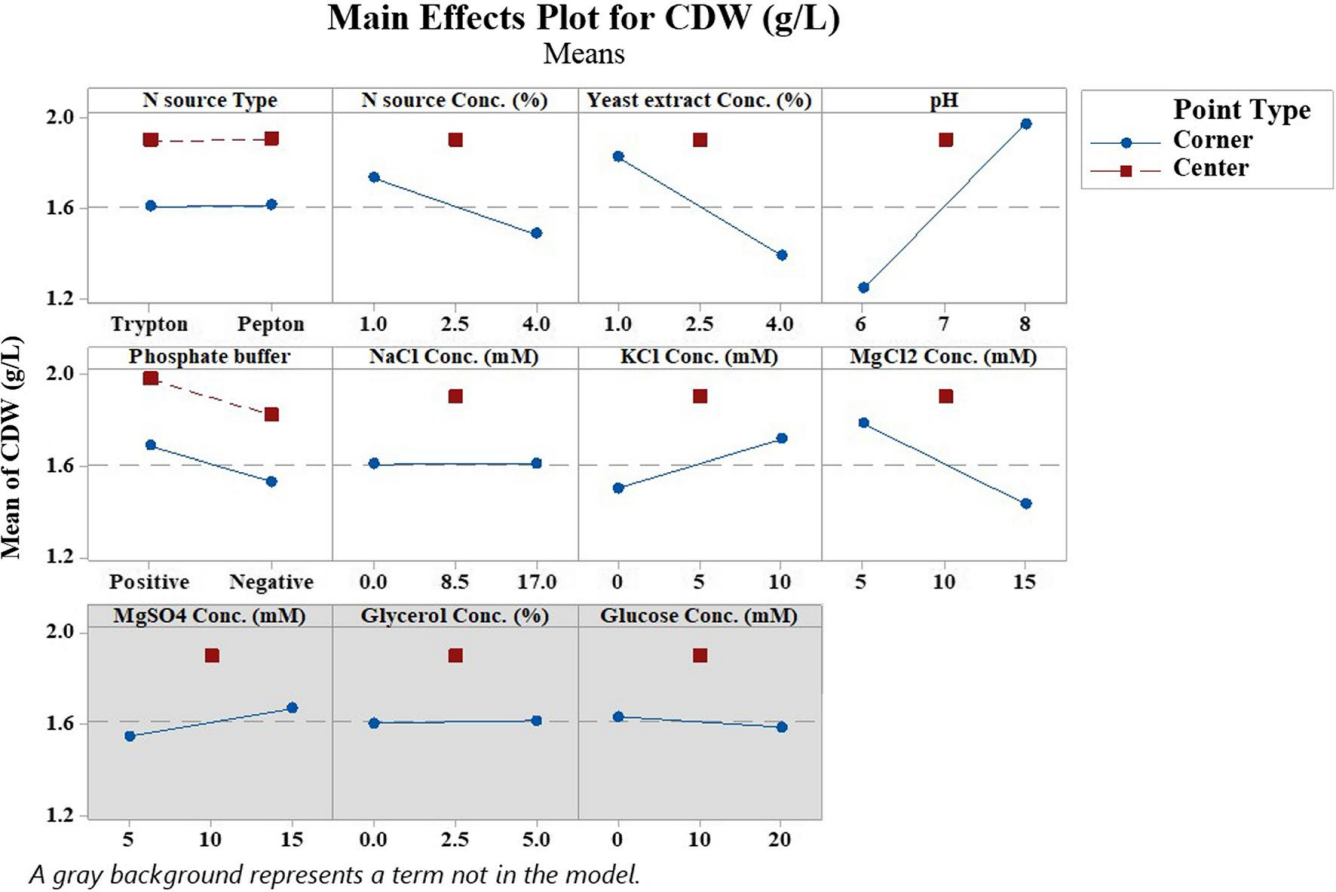
Main effects plot of screening experiment (PBD). Relative effect of each independent variable level on response mean is denoted

After data analysis, the model was significant with a *p*-value of 0.0 and an R^2^ of 92.96% (Table 2). Model terms including pH, Yeast extract, MgCl_2_, N source, and KCl concentration were effective factors with *p*-values less than 0.05. The higher F-value of a term corresponds to the higher association of the term and the response. Pareto chart (Fig. 1) is a graphical representation of the standardized effect of each variable on response. Reference line with the value of 2.228 denotes effectiveness of factors with larger values based on significance level (α = 0.05). According to this chart, the first 3 bars with larger values corresponding to pH, Yeast extract, and MgCl_2_ concentration were selected for optimization experiment design by the CCD method of RSM. Besides, to interpret the effect of each independent variable on the Response Mean, the Main effects plot was generated by Minitab software (Fig. 2). Nearly horizontal lines correspond to insignificant variables denoting that responses are affected by none of the factor’s levels. According to this plot, Tryptone was applied in optimization experiments as N source since there was no preference between Tryptone and Peptone. The media was supplemented by the center point level of Tryptone and KCl. Also, the central point concentration of NaCl and 0.89 mM phosphate buffer were added to the medium due to their slight refinement on the response mean. Glycerol, glucose, and MgSO_4_ were omitted from the model.

**Table 2:** ANOVA table of screening experiment narrating factors’ significance on SHuffle T7 growth

Source	Degree of freedom	Adjusted sums of squares	Adjusted mean squares	F value	*p* value
Model	9	3.35	0.37	14.67	0.0
Linear	8	2.95	0.37	14.5	0.0
N source type	1	0.0	0.0	0.01	0.935
N source conc. (%)	1	0.18	0.18	7.1	0.024
Yeast extract conc. (%)	1	0.56	0.56	22.12	0.001
pH	1	1.57	1.57	61.95	0.0
Phosphate buffer	1	0.12	0.12	4.96	0.05
NaCl conc. (mM)	1	0.0	0.0	0.00	0.989
KCl conc. (mM)	1	0.14	0.14	5.38	0.043
MgCl_2_ conc. (mM)	1	0.37	0.37	14.48	0.003
Curvature	1	0.41	0.41	16.07	0.002
Error	10	0.25	0.02		
Lack-of-fit	6	0.16	0.03	1.07	0.498
Pure error	4	0.1	0.02		
Total	19	3.61

#### Optimization by response surface methodology central composite design

The Design-Expert software generated 20 experiments for RSM-based optimization of chosen model terms, including pH, the concentration of Yeast extract, and MgCl_2_. Experiment runs were carried out in 50 mL culture containing a constant concentration of 2.5% Tryptone, 8.5 mM NaCl, 5 mM KCl and 0.89 mM Phosphate buffer in addition to varied values of Yeast extract, MgCl_2_, and pH according to each design point. Corresponding results were reported in the response column of CCD as presented in Table 3.

**Table 3:** Generated experimental runs for factor optimization via CCD and corresponding responses

Run	A: pH	B: Yeast extract (%)	C: MgCl_2_ (mM)	CDW (g/L)
1	8	4	15	1.8
2	8	1	15	2.2
3	7	5	10	1.4
4	8	1	5	1.9
5	6	4	15	1.2
6	7	2.5	10	2.1
7	7	2.5	10	2.1
8	8.6	2.5	10	2.8
9	7	2.5	18	1.7
10	5.3	2.5	10	1.5
11	7	2.5	1.6	1.9
12	6	1.0	15	1.5
13	7	0	10	1.3
14	7	2.5	10	2.1
15	7	2.5	10	2.3
16	7	2.5	10	2.2
17	6	4	5	1.3
18	6	1	5	1.5
19	8	4	5	2.0
20	7	2.5	10	2.2

After performing analyses by different models, the quadratic model was suggested to predict and validate the optimal condition. The model *p*-value was significant (0.0001), while its lack of fit was insignificant (0.1247) in proportion to the pure error, implying that error does not have any impact on the suggested model (Table 4). The R^2^ value of 0.9581, adjusted R^2^ of 0.9204, and predicted R^2^ of 0.7309 (Difference < 0.2) indicated a reasonable fitness of the model to the experimental data and can explain 95.8% of response variations. Besides, the adequate precision value (17.8198) indicates a sufficient signal, and a smaller value of PRESS (0.8345) than the total sum of squares (3.2) depicted that the model was fitted sufficiently.

**Table 4:** ANOVA table of culture media optimization for SHuffle T7 growth (Quadratic model)

Source	Sum of Squares	*df*	Mean Square	F-value	*p* value	
Model	3.06	9	0.3404	25.43	< 0.0001	Significant
A-pH	1.47	1	1.47	109.80	< 0.0001	
B-Yeast Extract	0.0353	1	0.0353	2.63	0.1356	
C-MgCl_2_	0.0070	1	0.0070	0.5258	0.4850	
AB	0.0002	1	0.0002	0.0187	0.8940	
AC	0.0051	1	0.0051	0.3846	0.5490	
BC	0.0586	1	0.0586	4.38	0.0629	
A^2^	0.0120	1	0.0120	0.8927	0.3670	
B^2^	1.31	1	1.31	98.07	< 0.0001	
C^2^	0.2779	1	0.2779	20.76	0.0010	
Residual	0.1339	10	0.0134			
Lack of Fit	0.1006	5	0.0201	3.03	0.1247	Not significant
Pure Error	0.0332	5	0.0066			
Cor Total	3.20	19				

**Fig. 3:**
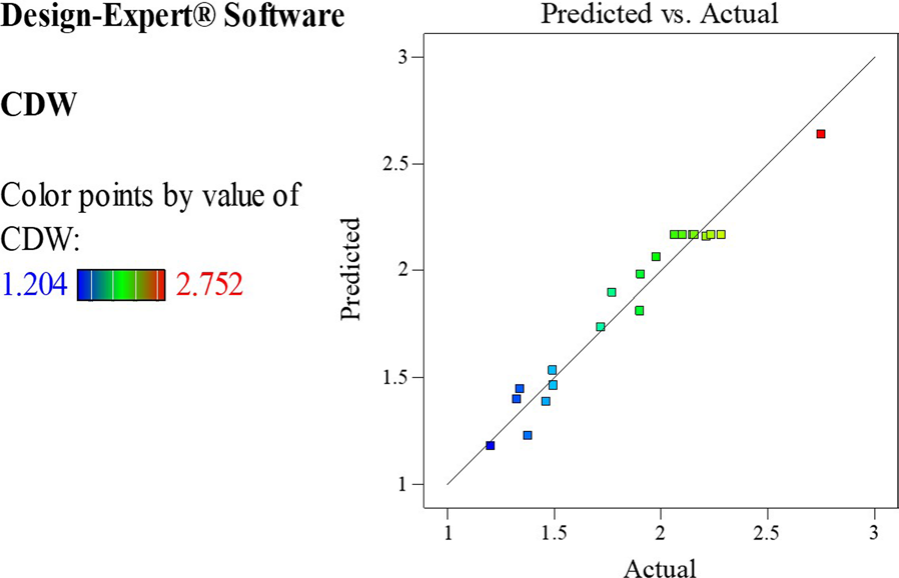
Predicted vs. Actual diagnostic plot. Graph of Predicted response values versus Actual response values of experiment runs generated by quadratic model

**Fig. 4:**
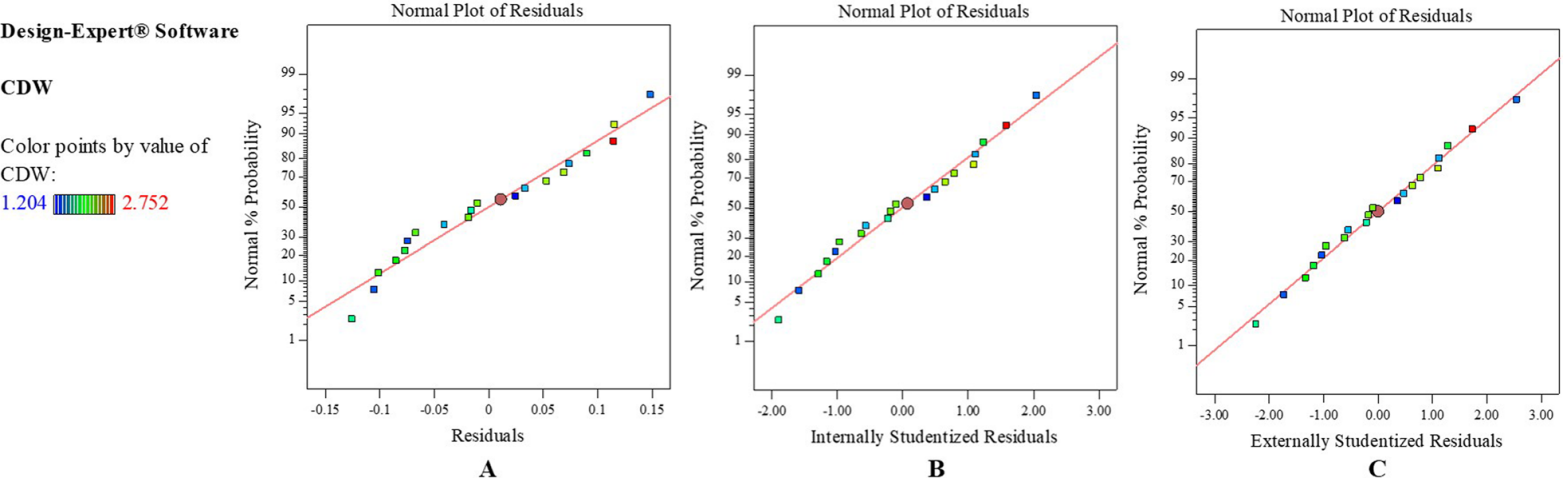
Residuals Normal probability diagnostic plots generated by quadratic model. **A** Normal probability plot of residuals. **B** Normal probability plot of externally Studentized residuals. **C** Normal probability plot of internally Studentized residuals

The goodness of fit of the quadratic model was further evaluated by diagnostic analyses that indicated the normality of data. The Predicted vs. Actual diagnostic plots denote that the actual response values of experiment runs were in acceptable agreement with predicted response values (Fig. 3). The compliance of the residuals with predicted values is illustrated in the Normal probability plots (Fig. 4). The Normal probability plots were linear and revealed that responses followed normal probability distribution, such that the residuals were in accordance with predicted values, and the model provided acceptable analyses.

**Fig. 5:**
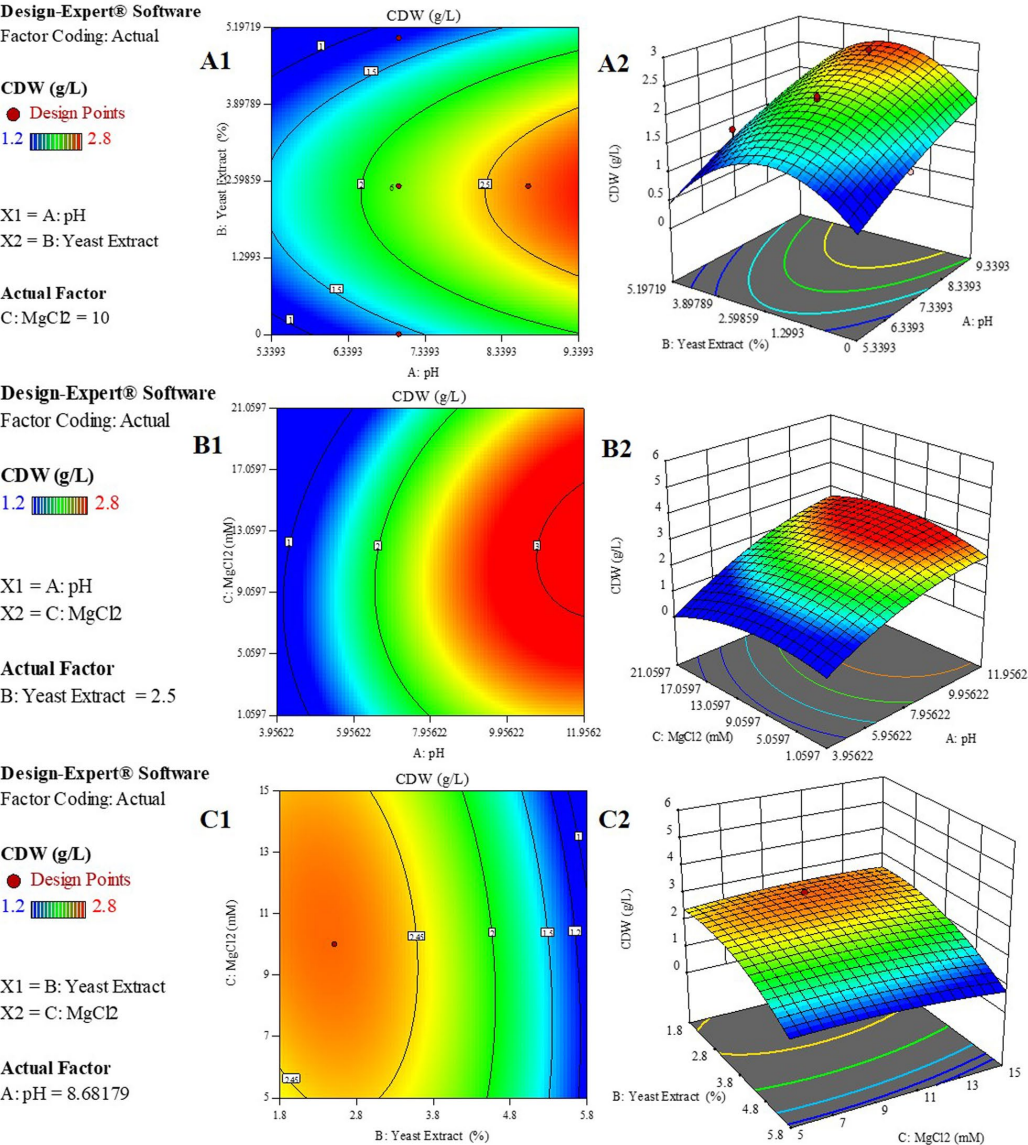
Contour (Left column) and 3D (Right column) plots of significant factors based on quadratic model. **A1, A2** Representing AB interaction when C is constant. **B1, B2** Representing AC interaction when B is constant. **C1, C2** Representing BC interaction when A is constant. Blue color indicates the lowest response yield while the red color shows the highest value of response

Terms with *p*-values less than 0.05 are considered significant, and thus, can affect the response parameters; therefore, A (pH) and quadratic effect of terms B (Yeast extract) and C (MgCl_2_), (B^2^ and C^2^) were significant model terms. Based on the quadratic model, the 3D and contour plots were generated (Fig. 5). According to Fig. 5, the highest response was accomplished when the media was supplemented by medium levels of Yeast extract (2.5%) and MgCl_2_ (10 mM) coupled with maximum pH (8).

The equation in terms of actual factors was achieved from the quadratic model depicting the mathematical model for biomass production with culture optimization process:

$$\begin{aligned} CDW \left( \frac{g}{L} \right) & = - 2.68893 + 0.67111\;pH + 0.736996\;Yeast\;Extract \\ & \quad + 0.099864\;MgCl_{2} + 0.003752\;pH * Yeast\;Extract \\ & \quad + 0.005074\;pH * MgCl_{2} - 0.011486\;Yeast\;Extract*MgCl_{2} \\ & \quad - 0.028798\;pH^{2} - 0.135958\;Yeast\;Extract^{2} - 0.00555\;MgCl_{2}^{2} \\ \end{aligned}$$

The Design-Expert software utilizes the obtained equation for point prediction according to chosen circumstances for each model term and response. Optimization was validated by examining three of the software suggestions with the highest desirability (Table 5). All resulted in an approximately same cell density of 2.5 g/L.

**Table 5:** Predicted optimal conditions for maximum Biomass production

No	pH	Yeast extract concentration (%)	MgCl_2_ concentration (mM)	CDW (g/L)	Desirability
1	8	2.5	10	2.468	0.963
2	8	2.5	9.5	2.465	0.963
3	8	2.4	10.2	2.468	0.963

**Fig. 6:**
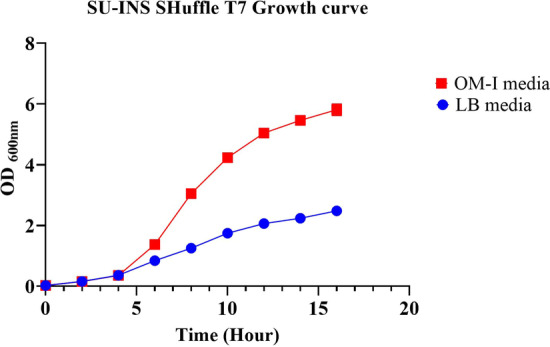
SU-INS SHuffle T7 growth curve in basic and optimized condition. Growth in LB media (Blue) and OM-I media (Red)

The optimum condition for maximum growth determined to be 2.5% Tryptone, 2.5% yeast extract, 10 mM MgCl_2_, 5 mM KCl, 8.5 mM NaCl and pH 8. The OM-I media was compared to LB media that resulted in more than 2.3-fold higher biomass with OD_600_ of approximately 5.8 (corresponding to 2.5 g/L CDW) compared to LB media (OD_600_ of 2.5 or 1.08 g/L CDW). The growth curve of SHuffle T7 culture in OM-I media was graphed against basic conditions (Fig. 6).

**Fig. 7:**
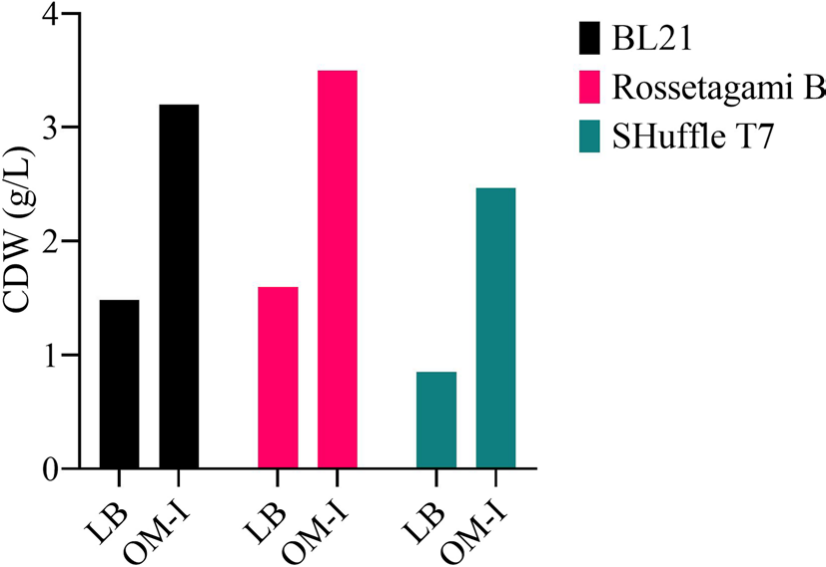
Evaluation of biomass production in OM-I compared to LB media for three *E. coli* strains holding SU-INS construct

OM-I was applicable for other *E. coli* strains including BL21 (DE3) and Rossetagami B holding similar gene construct (SU-INS). More than twofold biomass was obtained when cells were cultivated in OM-I media compared to LB media (Fig. 7).

#### Evaluation of optimal points for soluble expression in shake flask

**Fig. 8:**
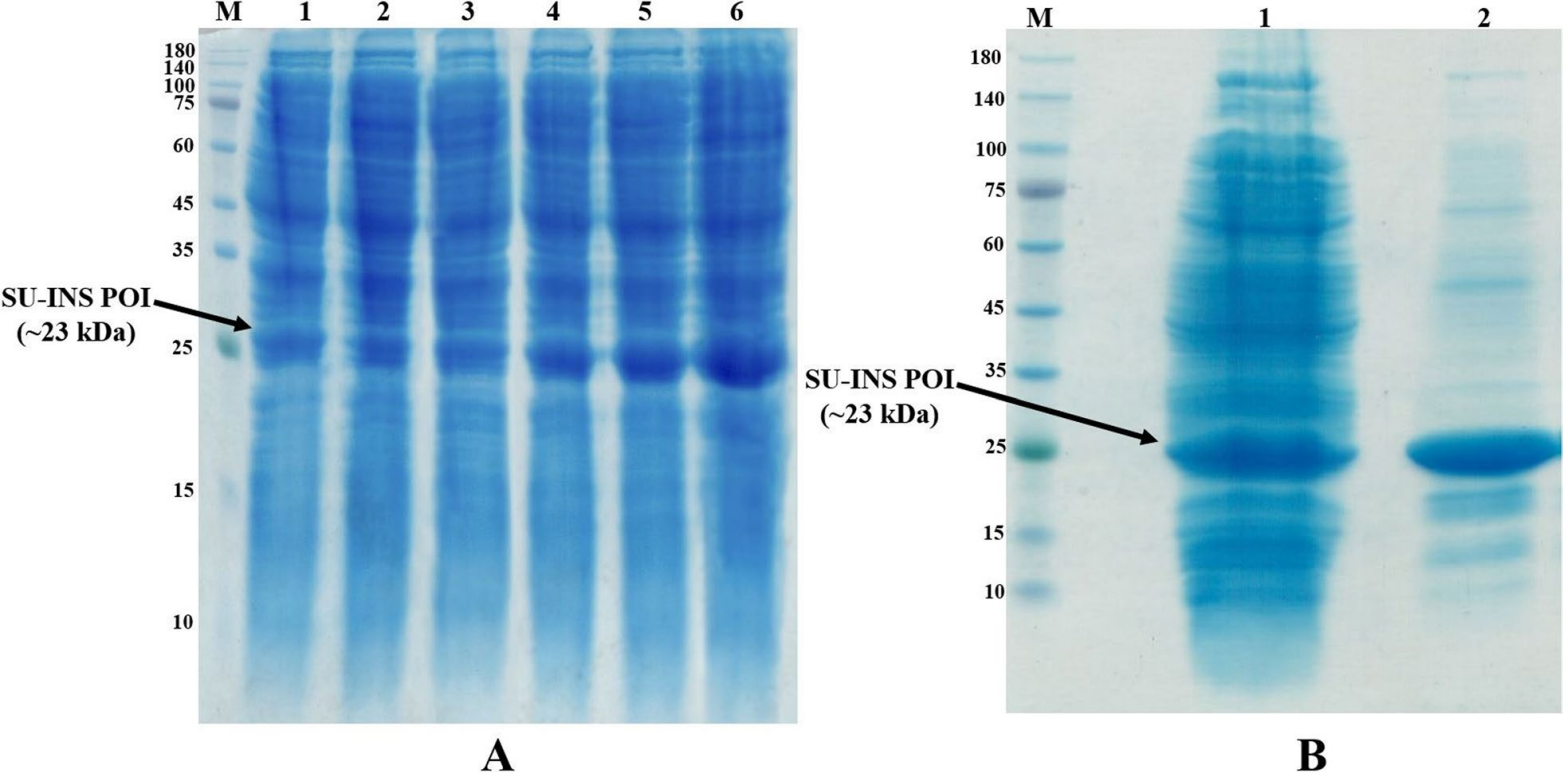
POI soluble expression and Purification. Coomassie stained 12% SDS-PAGE: **A** POI soluble expression in LB and OM-I media. M. Protein Marker. 1–3: POI soluble expression in LB media. 4–6: POI soluble expression in OM-I media. **B** SU-INS POI IMAC purification. M. Protein Ladder. 1: Cell lysate supernatant (Unpurified), 2: Purified POI

The soluble expression of the POI was evaluated in OM-I media compared to LB media in triplicates to assess the effect of media ingredient optimization on the soluble expression of the fusion protein. The results of experiments were visualized by Coomassie-stained SDS-PAGE that revealed competitively higher soluble POI produced in OM-I media (Fig. 8a).

#### Final product identity and bioactivity assessment

To evaluate the feasibility of bioactive Lispro insulin production from expressed fusion protein, the POI was purified, modified, and undergone proteolytic cleavage. His-tagged POI was isolated by Immobilized metal affinity chromatography (IMAC) via Nickel sepharose resin (Fig. 8b) [13].

The Purified POI was successfully converted to bioactive insulin Lispro and retained its solubility after the tag and C-peptide removal. The produced Lispro was identical to its commercially available analog considering electrophoretic mobility, LC–MS/MS, Circular Dichroism (CD), HPLC, and bioactivity analyses (Data not shown) [13].

#### Evaluation of OM-I media in fermentor (Batch culture)

**Fig. 9:**
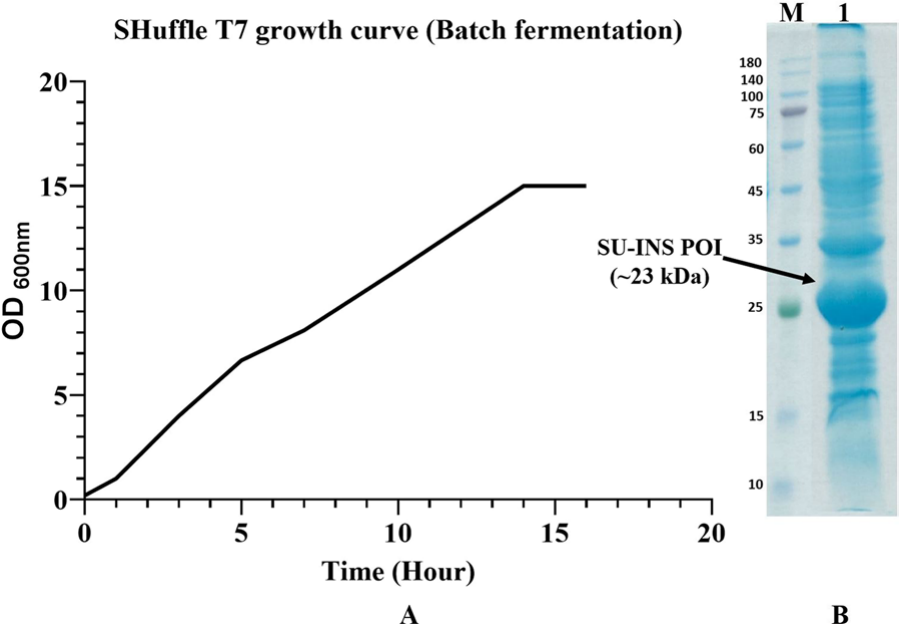
Evaluation of OM-I media in fermentor cultivation. **A** SU-INS SHuffle T7 growth curve during Batch fermentation. **B** SU-INS POI soluble expression in fermentor. Coomassie stained 12% SDS-PAGE: M. Protein Ladder. 1: Post-induction cell lysate supernatants

The large-scale applicability of optimal media was assessed in a 5 L volume fermentor vessel containing 3 L OM-I media. The final OD_600_ of 15 was achieved after 15 h of inoculation (8 h after induction), and bacterial culture went in the stationary phase at this point (Fig. 9a). Approximately 86 g bacterial wet weight corresponding to 6.45 g /L CDW was obtained after harvest. The bacteria pellet was resuspended in 35 mL of the Lysis buffer, and the soluble lysate was collected. SDS-PAGE results revealed a considerably high concentration of soluble POI obtained from fermentor culture (Fig. 9b) (Additional file 1; Fig. S1).

## Discussion

*E. coli* is one of the most employed hosts for recombinant protein production due to its advantageous characteristics such as rapid growth, easier genetic manipulations, and high yield recombinant protein synthesis rates [14]. *E. coli* was the first expression host used for manufacturing human insulin in 1982 [15]. However, due to its prone-to-aggregate structure, insulin expression in *E. coli* leads to IB formation [5]. We used the SUMO solubilizing tag and SHuffle T7 strain (SU-INS SHuffle T7 clone) in our previous work to prevent IB formation. Here in this study, we intended to optimize culture media composition to improve SU-INS SHuffle T7 growth rate and biomass yield.

The bacterial growth rate, similar to other natural processes, may have countless contributing parameters. Identification and optimization of these factors pose several challenges concerning expenditure and overall economics [16]. Considering this, DoE is a powerful tool in statistical bioprocess optimization that can obtain elevated results with reduced time and effort [17]. Several studies have utilized DoE methods to improve the yield of recombinant protein expression in *E. coli* through culture media optimization. Table 6 summarizes some of the previous similar studies considering product type, design method, evaluated factors, and the optimization outcome. L-Asparaginase, Phytase, Streptokinase, and Reteplase are some of the proteins expressed in *E. coli* and undergone DoE based culture media optimization that led to enhanced production yield. Based on reviewed literature (Table 6), numerous variables may affect bacterial growth rate and biomass production. The type and concentration of C and N source, pH, and trace elements are some of the most studied variables.

**Table 6:** Literature review on DoE-based optimization of *E. coli* culture condition

Host Organism	Product	Design	Studied factors	Product increase	Reference
*E. coli* SHuffle T7	Biomass (SU-INS)	PBD, CCD	C, N sources, inorganic salts, pH	2.3X (shake flask), 5.9X (fermentor)	Present study
*E. coli* BL21 (DE3)	Phytase	CCD	C, N sources, salt	1.2X	[18]
*E. coli* (ATCC no. 11303)	L-Asparaginase	OFAT, CCD	C, N sources, ions, inoculum age and size	10X	[19]
*E. coli* BL21 (DE3)	L-Asparaginase II	FFD, CCFD	C, N sources, ions	NA	[20]
*E. coli* BL21 (DE3)	Streptokinase	PBD, CCD	C, N sources, ions	7X	[21]
*E. coli*	Dihydrolipohyl Dehydrogenase	CCFD	C, N sources, inorganic salts, pH	1.2X (shake flask), 1.7X (fermentor)	[22]
*E. coli* DH5α	Rheumatoid arthritis DNA vaccine	OFAT, PBD, BBD	C, N sources, salts, trace elements, pH, inoculum size, temperature	51.9%	[23]
*E. coli* MET-3	L-Methionine	PBD, BBD	C, N sources, salts	1.5X (shake flask), 6.4X (fermentor)	[24]
*E. coli* BL21 (DE3)	Reteplase	BBD	Temperature, Agitation, pH	2X	[25]
*E. coli* Δwaaf	Colanic acid	FFD, CCD	C, N sources, inorganic salts	12X	[26]
*E. coli* BL21 (DE3)	γ-cyclodextrin glycosyltransferase	OFAT, PBD, Steepest ascent path, BBD	C, N sources, inorganic salts, pH	2.83X	[27]

In this study, PBD was used to screen the effect of eleven factors on cell growth including, the concentration of various N and C sources, salts, metal ions, pH, and the buffering system. Among mentioned factors, pH, Yeast extract, and MgCl_2_ concentration had the most influence on cell growth and, thus, were chosen for further optimization by RSM Central Composition Design. To a lesser extent, the concentration of N source and KCl was also significant such that their central point level led to a higher response (Fig. 2). Thus, their mid-point concentration was used in the culture media. Additionally, the presence of 0.89 mM Phosphate buffer was beneficial for cell growth; similarly, the central point value of NaCl corresponded to a high response mean, thus were used in culture media. Model terms with insignificant *p*-values, such as MgSO_4_, glycerol, and glucose concentrations, as well as the N source type, were omitted from media in CCD experiments.

According to RSM results, pH was the most influential factor as though its highest level correlated with higher cell growth and biomass. Our result was in agreement with other work which reported that a pH increase could improve the level of Reteplase production in *E. coli* [25]. Avoiding cellular stresses such as the metabolic burden of acidification and proteases during the synthesis of recombinant proteins can contribute enormously to overall cell growth [10]. C source metabolism leads to the accumulation of acetate and acidic by-products in the culture medium that can reduce cell growth and recombinant protein production. In this case, the addition of Yeast extract and Tryptone can prevent medium acidification due to the high amount of ammonia produced during their metabolism [10, 28]. Likewise, maintaining pH 8 in the culture medium ameliorates the acetate stress in *E. coli* cultivation [29]. OM-I is a suitable media by being rich in Yeast extract and Tryptone, in addition to the presence of a strong buffer (pH 8) that evokes elevated cell growth and delayed entrance to the death phase. The proposed media enhanced *E. coli* SHuffle T7 biomass by 2.3 fold in shake flask which further increased by an extra 2.6 fold in batch culture fermentor. OM-I is a suitable media for high cell density fermentation of other *E. coli* strains such as Rossetagami B and BL21 (DE3).

## Conclusion

The optimum cultivation medium composition was demonstrated for SU-INS SHuffle T7 clone expressing SUMO-Lispro proinsulin fusion protein. The optimal media (OM-I media) was validated and compared to basic media (LB media), which led to approximately 2.3 fold more biomass. The OM-I is an efficient media for the SU-INS fusion protein production in shake flask which is reproducible in large-scale fermentation.

## Methods

### Microorganism, culture media, chemicals and software

*E. coli* SHuffle T7 strain (purchased from NEB) transformed by pET21a + vector containing SU-INS construct (GenBank accession no. MW291010) was used in this study. SU-INS construct contained N-terminal 6XHis-tag and SUMO fusion tag. Luria- Bertani (LB) media used as the basic culture media for primary evaluation of growth and soluble expression of SU-INS SHuffle T7 clone. BL21 and Rossetagami B strains (purchased from Novagen) were applied as alternative host strains to assess the applicability of optimized media. Chemical ingredients were purchased from either Merck or Sigma. Protein weight marker (PS-103) was supplied from Jena. Minitab18.1.0 software (Minitab Inc., State College, PA, USA) was applied for screening experiments. Optimization experiments were designed and analyzed by Design-Expert 11.0.0 software (Stat-Ease, Inc., Minneapolis, MN, USA).

### Seed preparation for DoE

The newly transformed frozen stock of SU-INS SHuffle T7 was cultured on a streak plate and was incubated at 30 °C for 16 h to obtain single colonies. Then, 10 mL LB media in a 50 mL shake flask was inoculated by a single colony and incubated overnight at 30 °C and 180 rpm (revolutions per minute) shake until reaching the OD_600_ (Optical Density at λ = 600 nm) of 2. Afterward, seed culture was scaled up in a 500 mL volume shake flask containing 100 mL LB media by inoculating 2 mL of pre-culture and incubated at 30 °C with 180 rpm shaking. After reaching the OD_600_ of 2 bacterial culture was centrifuged at 2500xg for 5 min, and then cells were resuspended in 20 mL WFI (Water for injection) immediately before use.

### Cell dry weight measurement

Cell dry weight per 1 Liter of culture media (g/L CDW) was measured for 20 mL culture volume according to the method described by [30]. OD_600_ to CDW conversion coefficient was approximately 0.43 g. CDW was calculated via the multiplication of OD_600_ values by 0.43.

### Optimization of cultivation medium

Firstly, PBD screened the effectiveness of various factors. Then, the CCD method of RSM optimized the level of influential variables. All experiments were carried out in 250 mL volume shake flasks containing 50 mL culture media. Media was prepared according to each designed point and inoculated by seed culture to the initial OD_600_ of 0.1 and then incubated at 30 ͦ C with 180 rpm shake for 16 h. The OD_600_ of culture was used for measuring bacterial growth via Plate reader (Biotek SynergyHTX, USA). Then, CDW (g/L) was calculated as the response of experiments.

#### Factor screening via Plackett–Burman factorial design

Eleven factors examined in the screening experiment included the concentration of various N and C sources, pH, presence of 0.89 mM phosphate buffer, and the concentration of salts and metal ions. Twenty experiments, including eight central points (Table 1) designed by two-level Plackett–Burman factorial design via Minitab software. After performing experiments, responses were analyzed statistically. Model validation parameters and variable significance values were reported in ANOVA (Analysis of variance) and fit statistic tables. Significant variables (*p*-values < 0.05) were selected based on the ANOVA table, Pareto chart of standardized effects, and main effects plot of response means.

#### Factor optimization via response surface methodology

Based on PBD results, three of the most significant factors were selected for further optimization by 5-level CCD in Design-Expert software resulted in twenty experimental runs, including six central points (Table. 3). To prepare culture media for each run, the specified composition of model terms (chosen factors) were used according to designed points. Besides, constant values of other media components that were not in the model were supplemented in the media according to the Main effects plots of PBD. The concentration that corresponded to the highest response for less significant variables and -1 level of insignificant factors were supplied (Fig. 2). Following the execution of experiments, responses were analyzed via different models. The best model was selected based on model validation parameters reported in the ANOVA table and fit statistic tables in addition to diagnostic analysis. Design-Expert software generated the diagnostic reports and plots, including the Predicted vs. Actual diagnostic plot and Normal Probability plots of Residuals. The effect of each significant independent and dependent variable on response was reported graphically via contour and 3D plots. Finally, Design-Expert software generated predictions about optimal points based on the obtained regression equation. Predicted design points with the highest desirability were examined and compared to the basic condition (Cultured in LB media) in triplicates. The suggested optimal media was named OM-I media.OM-I media was examined for other *E. coli* strains, including BL21 (DE3) and Rossetagami B holding SU-INS construct compared to LB media and their biomass was measured.

### Soluble expression analysis

The expression of SUMO-Lispro proinsulin fusion protein was evaluated in OM-I media compared to LB media in triplicates to assess the efficacy of optimal media to express the protein of interest (POI) in soluble form. The experiments were carried out in 250 mL volume shake flasks containing 50 mL of either OM-I or LB media. Each shake flask was inoculated by seed culture to initial OD_600_ of 0.1 and then incubated at 30 °C with 180 rpm agitation until reaching the OD_600_ of 0.6. Then, cultures were induced by 0.4 mM IPTG and were incubated at 30 °C for 8 h. Cultures were centrifuged at 8000xg for 20 min. The obtained pellet of each experiment was resuspended in 5 mL Lysis buffer (50 mM NaH_2_PO_4_, 300 mM NaCl, 10% Glycerol, 1 mM PMSF, pH 8), sonicated (10 bursts of 30 s followed by 1-min rest after each interval), and centrifuged at 15000xg for 30 min. The supernatant of each experiment was collected. Soluble expression of POI was assessed by 12% SDS-PAGE.

### POI purification and bioconversion

The attainability of properly folded Lispro insulin was assessed through POI isolation and proteolytic conversion according to protocols explained in our submitted manuscript [13]. Purification of His-tagged POI was achieved through Nickel sepharose resin. Purification efficiency was assessed by SDS-PAGE and visualized by Coomassie blue staining. POI was converted to Lispro insulin by Trypsin and Carboxypeptidase B cleavage. Lispro was purified by Source™ 30RPC resin. The identity of the final product was evaluated by electrophoresis, LC–MS/MS, RP-HPLC, CD analyses, and bioactivity compared to commercial Lispro insulin as reference [13].

### Fermentor cultivation and expression

Batch culture fermentation was carried out to assess the reproducibility of optimized culture media for larger scales. Fermentor seed pre-culture was prepared in 15 mL OM-I media containing 50 µg/mL Ampicillin and incubated at 30 °C until reaching the OD_600_ of 2. Afterward, pre-culture was scaled up in a 2 L shake flask containing 300 mL OM-I media generating the initial OD_600_ of 0.1. Then, seed culture was incubated at 30 °C with 180 rpm agitation until reaching the OD_600_ of 2 and was used as fermenter seed. 2.7 L OM-I media was prepared and applied into a 5 L fermentation vessel (New Brunswick Scientific Co., USA). 300 mL seed was added to the fermentor vessel to obtain the initial OD_600_ of 0.2. Fermentation was carried out at 30 °C, and the acidity of culture was maintained at pH 8 by Ammonia solution. DO (Dissolved oxygen) was set constant at 37%, and aeration was set at 1 vvm (Volume of air per unit of medium per unit of time (L/L/m)), and agitation was controlled by DO changes to a maximum of 800 RPM. Samples were collected each one hour until reaching the OD_600_ of 6. At this point, the culture was induced by 0.4 mM IPTG. After induction, growth was monitored hourly until the beginning of the stationary phase. Bacterial culture was harvested by centrifugation at 4500xg for 45 min. Bacterial pellet resuspended in Lysis buffer (5 mL/g bacterial wet weight) and homogenized at 600 psi twice. Then, the homogenized cell lysate was centrifuged, and its supernatant was collected. Soluble expression of POI was assessed by 12% SDS-PAGE.

## Supplementary Information

**Additional file 1: Figure S1**. Original Figure 8A. POI soluble expression and Purification. Coomassie stained 12% SDS-PAGE: POI soluble expression in LB and OM-I media. M. Protein Marker. 1–3: POI soluble expression in LB media. 4–6: POI soluble expression in OM-I media. **Figure S2**. Original Figure 8B. POI soluble expression and Purification. Coomassie stained 12% SDS-PAGE: SU-INS POI IMAC purification. M. Protein Ladder.1: Cell lysate supernatant (Unpurified), 2: Purified POI. **Figure S2**. Original Figure 9B. SU-INS POI soluble expression in fermentor. Coomassie stained 12% SDS-PAGE: M. Protein Ladder.1: Post-induction cell lysate supernatants

## Data Availability

The datasets supporting the conclusions of this article are included within the article.

## Abbreviations

IB: Inclusion body
*E. coli*: *Escherichia coli*
RSM: Response surface methodology
PBD: Plackett–Burman design
CCD: Central composite design
SUMO: Small ubiquitin-related modifier
trxB: Thioredoxin reductase gene
gor: Glutathione reductase gene
DsbC: Disulfide bond isomerase
OFAT: One-factor-at-A-time
DoE: Design of experiment
ANOVA: Analysis of variance
PRESS: Predicted residual error sum of squares
SU-INS: 6XHis-SUMO tag-Lispro proinsulin construct
FFD: Fractional factorial design
CCRD: Central composite rotatable design
BBD: Box–Behenken design
CCFD: Central composite face-entered design
OM-I: Optimized media-I
IPTG: Isopropyl β-d-1-thiogalactopyranoside
OD: Optical density
PMSF: Phenylmethylsulfonyl fluoride
POI: Protein of interest
SDS-PAGE: Sodium dodecyl sulfate–polyacrylamide gel electrophoresis
LB: Luria–Bertani
psi: Pounds per square inch
DO: Dissolved oxygen
RPM: Revolutions per minute
VVM: Volume of air per unit of medium per unit of time
CDW: Cell dry weight
