# Supplementary material for: Media optimization for SHuffle T7 Escherichia coli expressing SUMO-Lispro proinsulin by response surface methodology
Source: BMC Biotechnol. 2022 Jan 3;22:1. doi: 10.1186/s12896-021-00732-4 (PMC8722112; doi:10.1186/s12896-021-00732-4)
Supplement: Supplementary file 1 — Additional file 1: Figure S1. Original Figure 8A. POI soluble expression and Purification. Coomassie stained 12% SDS-PAGE: POI soluble expression in LB and OM-I media. M. Protein Marker. 1–3: POI soluble expression in LB media. 4–6: POI soluble expression in OM-I media. Figure S2. Original Figure 8B. POI soluble expression and Purification. Coomassie stained 12% SDS-PAGE: SU-INS POI IMAC purification. M. Protein Ladder.1: Cell lysate supernatant (Unpurified), 2: Purified POI. Figure S2. Original Figure 9B. SU-INS POI soluble expression in fermentor. Coomassie stained 12% SDS-PAGE: M. Protein Ladder.1: Post-induction cell lysate supernatants [file 12896_2021_732_MOESM1_ESM.pptx]

## Slide 1
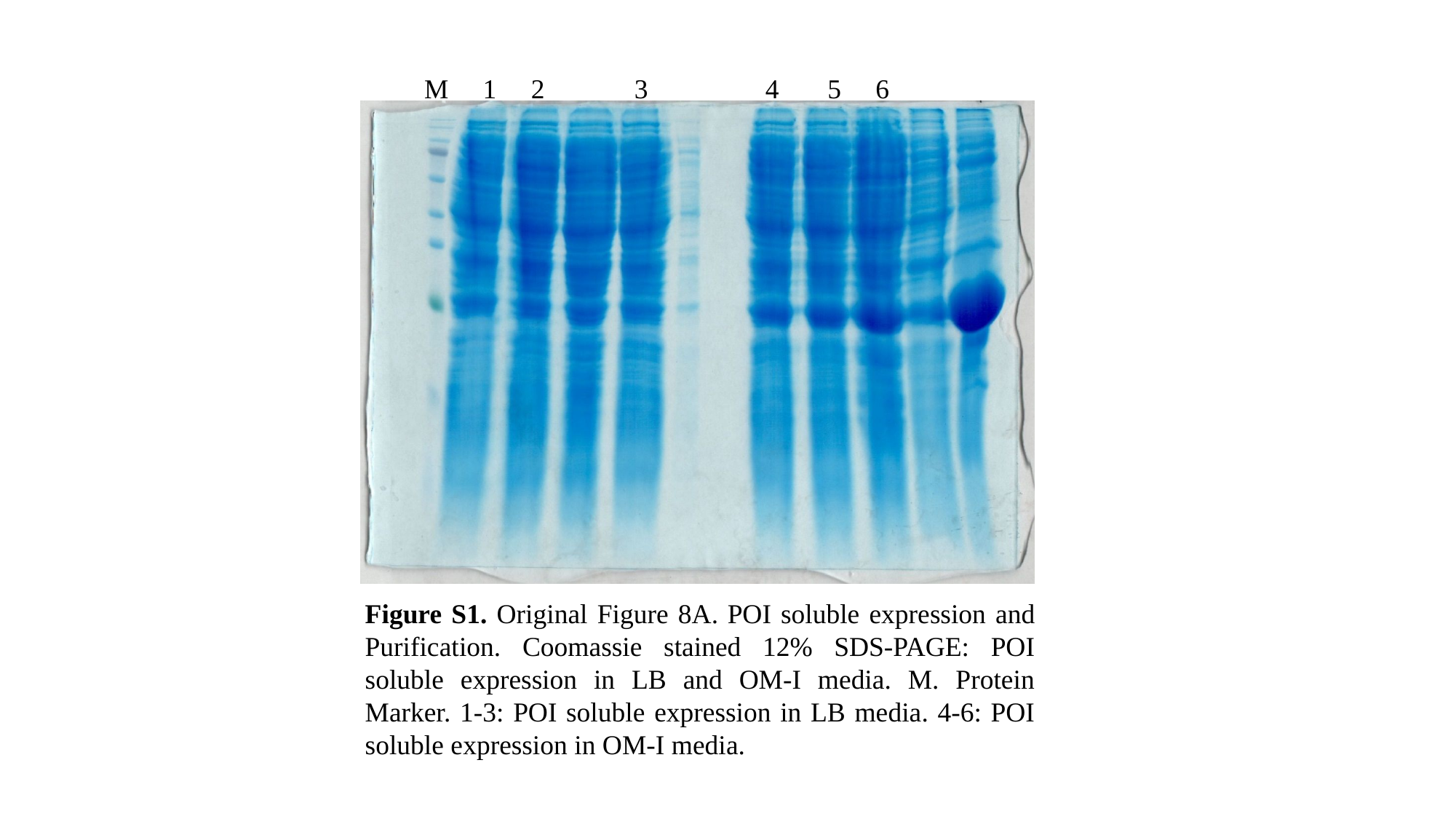

M 1 2 3 4 5 6
Figure S1. Original Figure 8A. POI soluble expression and Purification. Coomassie stained 12% SDS-PAGE: POI soluble expression in LB and OM-I media. M. Protein Marker. 1-3: POI soluble expression in LB media. 4-6: POI soluble expression in OM-I media.

## Slide 2
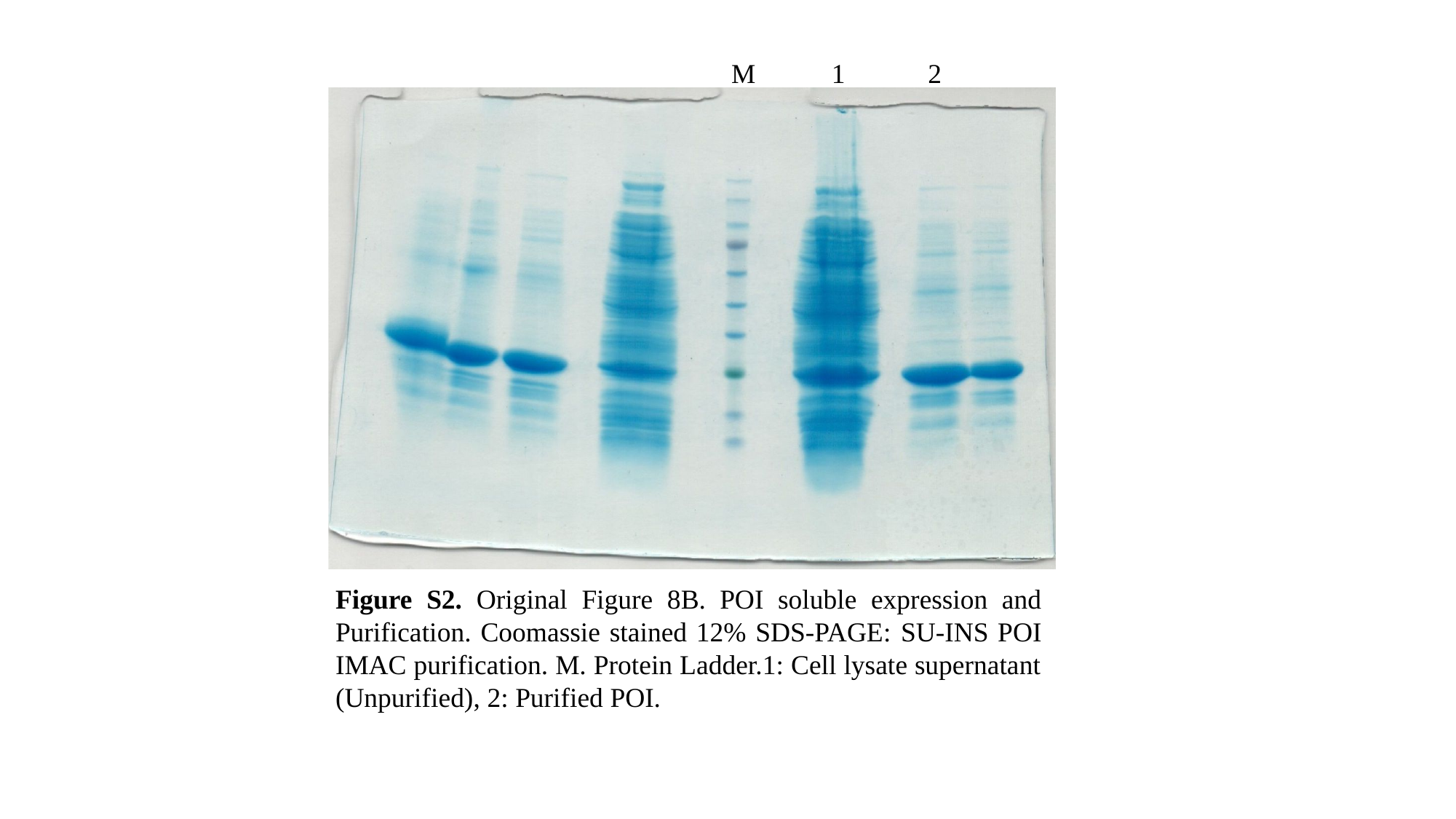

M 1 2
Figure S2. Original Figure 8B. POI soluble expression and Purification. Coomassie stained 12% SDS-PAGE: SU-INS POI IMAC purification. M. Protein Ladder.1: Cell lysate supernatant (Unpurified), 2: Purified POI.

## Slide 3
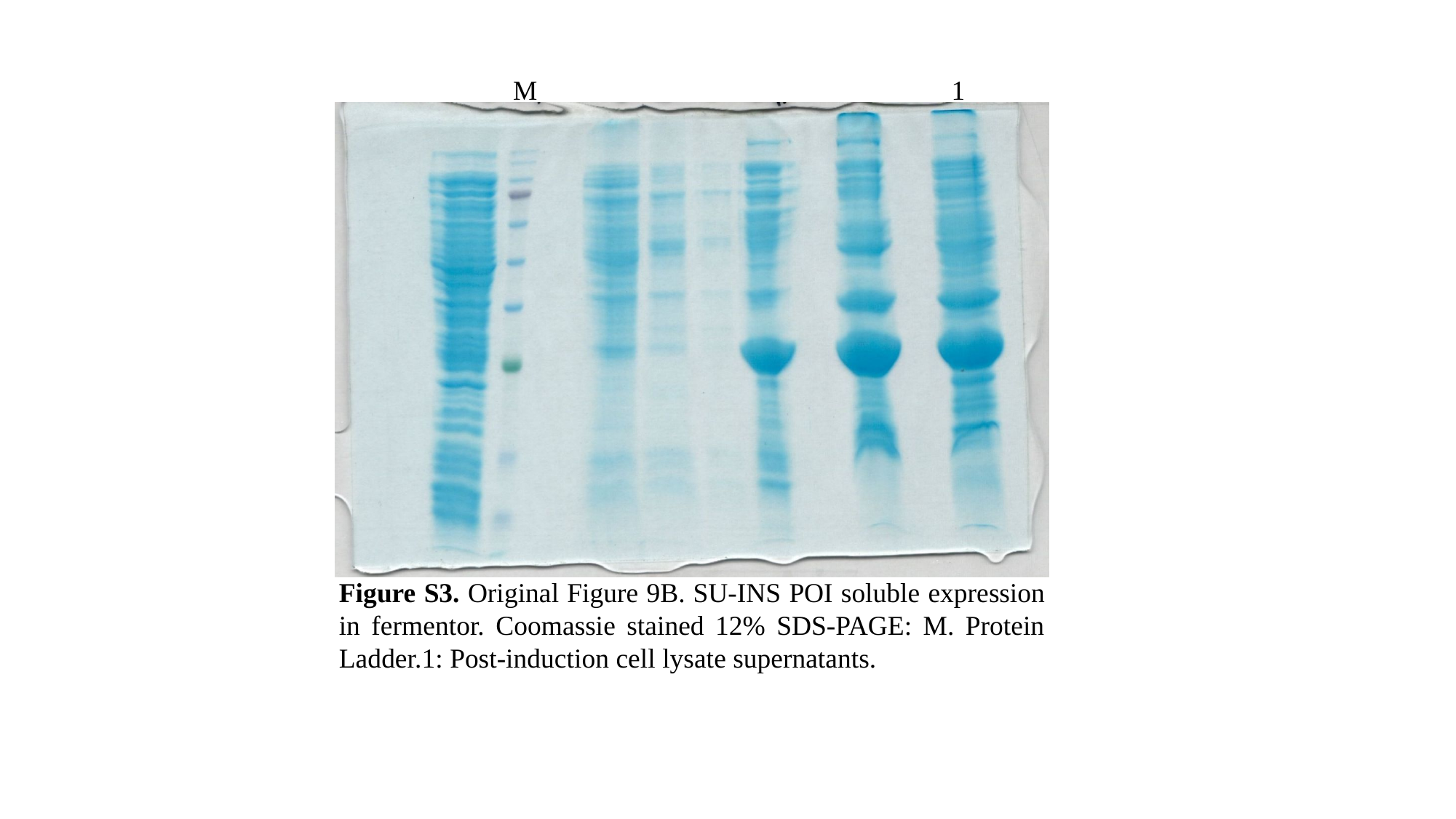

M 1
Figure S3. Original Figure 9B. SU-INS POI soluble expression in fermentor. Coomassie stained 12% SDS-PAGE: M. Protein Ladder.1: Post-induction cell lysate supernatants.
